# Outcome of 1939 traumatic brain injury patients from road traffic accidents: Findings from specialist medical reports in a low to middle income country (LMIC)

**DOI:** 10.1371/journal.pone.0284484

**Published:** 2023-09-13

**Authors:** Justina Teh, Mazlina Mazlan, Mahmoud Danaee, Ria Johanna Waran, Vicknes Waran

**Affiliations:** 1 Department of Rehabilitation Medicine, Faculty of Medicine, Universiti Malaya, Kuala Lumpur, Malaysia; 2 Department of Rehabilitation Medicine, Hospital Tuanku Ja’afar Seremban, Seremban, Negeri Sembilan, Malaysia; 3 Department of Social and Preventive Medicine, Faculty of Medicine, Universiti Malaya, Kuala Lumpur, Malaysia; 4 Department of Surgery, Division of Neurosurgery, Faculty of Medicine, Universiti Malaya, Kuala Lumpur, Malaysia; Duke University Medical Center: Duke University Hospital, UNITED STATES

## Abstract

**Objective:**

Road traffic accident (RTA) is the major cause of traumatic brain injury (TBI) in developing countries and affects mostly young adult population. This research aimed to describe the factors predicting functional outcome after TBI caused by RTA in a Malaysian setting.

**Methods:**

This was a retrospective cross-sectional study conducted on specialist medical reports written from 2009 to 2019, involving patients who survived after TBI from RTA. The functional outcome was assessed using the Glasgow Outcome Scale-Extended (GOSE). Factors associated with good outcome were analysed via logistic regression analysis. Multivariate logistic regression analysis was used to derive the best fitting Prediction Model and split-sample cross-validation was performed to develop a prediction model.

**Results:**

A total of 1939 reports were evaluated. The mean age of the study participants was 32.4 ± 13.7 years. Most patients were male, less than 40, and with average post RTA of two years. Good outcome (GOSE score 7 & 8) was reported in 30.3% of the patients. Factors significantly affecting functional outcome include age, gender, ethnicity, marital status, education level, severity of brain injury, neurosurgical intervention, ICU admission, presence of inpatient complications, cognitive impairment, post-traumatic headache, post traumatic seizures, presence of significant behavioural issue; and residence post discharge (p<0.05). After adjusting for confounding factors, prediction model identified age less than 40, mild TBI, absence of post traumatic seizure, absence of behaviour issue, absence of cognitive impairment and independent living post TBI as significant predictors of good functional outcome post trauma. Discrimination of the model was acceptable (C-statistic, 0.67; p<0.001, 95% CI: 0.62–0.73).

**Conclusion:**

Good functional outcome following TBI due to RTA in this study population is comparable to other low to middle income countries but lower than high income countries. Factors influencing outcome such as seizure, cognitive and behavioural issues, and independent living post injury should be addressed early to achieve favourable long-term outcomes.

## Introduction

Traumatic brain injury (TBI) is a critical public health issue that can lead to a large number of impairments contributing to irreversible disabilities [1]. Over the years, many studies have examined the functional outcome of patients after TBI [2–10], however the wealth of the data in the literature were mostly from developed countries. Differences in outcome has been previously highlighted, where patients from low to middle income countries (LMIC) having worse early prognosis compared to patients from high income countries (HIC) [11]. Also of note, the strength of association between some predictors and outcome differed between region, with low Glasgow Coma Scale (GCS) score predicting worse outcome in LMIC whereas increasing age had worse prognosis in HIC [11].

The discrepancy in outcomes following TBI between HIC and LMIC emphasized the importance of accumulating data from the LMIC. Most of the studies available in LMIC, especially in Southeast Asia, were small, with limited sample size and short duration of follow up [12–14]. One large TBI study in Vietnam with a long term follow up beyond 40 years, has examined the outcome of 1221 veterans with penetrating brain injuries. The study provided good data on various aspects affected by TBI [15], but the study population does not represent majority of TBI survivors in that country.

RTA, especially from motorcycle collision, is still the major cause of TBI in LMIC in Southeast Asia including Malaysia. In 2014, injury was the fifth most common cause of hospitalisation in the public hospitals in Malaysia, with RTA contributing 80% of trauma cases [16]. There is an increasing number of annual RTA cases in Malaysia and affected 1.74% of the population every year [17]. The Malaysian National Trauma Database reported that RTA mainly affecting younger male populations aged 15 to 24 years old [18]. Because of its high association with multiple concomitant injuries, TBI from RTA resulted in highly variable outcomes, despite the severity of the brain injury sustained [19–21].

With the advancement of acute care post TBI, the number of survivors post TBI has increased in recent years compared to the fatalities [22]. The survivors may have persistent impairments that impede participation and social integration years after TBI [2, 7, 10], hence the importance of understanding the functional outcome and the factors associated with the outcome in the local context. A recent systematic review described TBI research in Malaysia as still in its infancy state, with multiple limitations and lacking in population TBI awareness [23].

Since RTA is the main cause of TBI in Malaysia and affecting mostly young adult population, the main objective of this study was to examine the sociodemographic and clinical factors associated with functional outcome in patients with TBI from RTA. Previous studies have also noted discrepancies in functional outcome between different races, with black and minority groups faring worse [24, 25]. Malaysia is one of the LMICs with three major different ethnicities (Malay being the predominant ethnicity, followed by Chinese and Indian), thus it would be beneficial to understand how ethnicities play a role in the outcome of these patients too.

## Methods

The present study was approved by the University Malaya Medical Centre (UMMC) Medical Research Ethics Committee, with registration number 2021118–9725. This was a retrospective cross-sectional study using universal sampling, conducted by reviewing all the available specialist medical reports from 2009 to 2019, for patients who sustained TBI following RTA in Malaysia. The data were fully anonymized before the authors accessed them and the ethics committee waived the requirement for informed consent due to its retrospective nature.

These reports were written by a senior medical consultant in UMMC (VW) for medical insurance claimant purposes. Inclusion criteria for the study were Malaysian citizens, aged 18 and above at the time of injury, and involved in the first episode of TBI following RTA. Patients with previous history of TBI or other medical illnesses with pre-existing significant cognitive, behavioural and physical disabilities were excluded.

Data obtained from the reports were demographic data, injury details, complications and detailed function at home and in the community. The demographic data include age, gender, ethnicity, preinjury marital status, and premorbid education level. Marital status was defined as married, single or divorced. Premorbid education level was defined as no education, completed primary education, secondary education or tertiary education.

Injury details such as the vehicle involved in RTA, severity of TBI, duration post TBI, neurosurgical intervention, presence of concomitant injuries including injuries to the neck, face, chest, abdomen, spine, upper and lower extremities, presence of medical complications after injury including headache, seizures, pain, behavioural issue, cognitive and physical impairment were also obtained from the medical reports. Other information such as acute inpatient complications such as hospital acquired infections, surgical wound infections and pressure ulcers, subsequent admissions, and residence after discharge were analysed as well.

Severity of TBI was based on the initial GCS upon presentation to hospital. GCS of 13–15 were categorized as mild TBI, 9–12 were categorized as moderate TBI, while severe TBI were GCS of 8 or less. Duration post TBI was calculated from the initial event of injury to the time of specialist review, subcategorized into less than 2 years, 2 to 5 years, and more than 5 years. Presence of significant behavioural issues which include aggression, isolation and severe mood disorders were documented using the subjective explanation from the patient or family members, when they caused disturbances to daily functions, communication, family relationship or return to community. Cognitive impairment in the report was assessed using Mini Mental State Examination (MMSE). A score of more than 24 is normal, whereas a score of 20 to 24 is mild, 13 to 19 is moderate, and 12 and below is severe impairment.

Residence post discharge was classified as staying alone or with friends, with own immediate family such as spouse or children, with parents, or nursing home. Salary preinjury were further subcategorized based on income classification in Malaysia: B40 (bottom 40% of Malaysian income) which was less than RM4850 per month, M40 (Middle 40% group) of income from RM4850 to RM10959, and T20 (Top 20% group) of income more than RM10960 [26].

The functional status of each patient was then categorized using the Glasgow Outcome Scale-Extended (GOSE). GOSE consists of 8 categories which are: death (Category 1), vegetative state (category 2), lower severe disability (category 3), upper severe disability (category 4), lower moderate disability (category 5), upper moderate disability (category 6), lower good recovery (category 7) and upper good recovery (category 8). Structured GOSE interview form was used to guide the scoring (reference). In this study, the functional outcomes were divided into two groups: ‘good’ and ‘poor’ outcomes, using similar approaches as in other studies to ease bivariate and multivariate analyses [21, 23]. Good outcome includes patients in the upper and lower good recovery group which is category 7 and 8 in the GOSE scale. Patients in these categories do not require any assistance in activity of daily living and are able to engage in vocational and life activities. Poor outcome consists of patients in category 2 to 6.

Based on the framework described by Steyerberg and colleagues, a model performance assessment was designed [27]. We developed a prediction model from this cohort of patients and model performance was assessed in derivation and validation data sets. The derivation cohort included 1378 patients randomly allocated from the 1939 patients. The validation cohort consisted of the remaining 561 patients from the whole sample. Multivariate logistic regression analysis was used to derive the best fitting prediction model. The model was then internally validated using split-sample cross-validation with the validation cohort.

### Statistical analysis

The analysis was performed in two phases. The first phase was to determine the factors associated with functional outcome. The continuous variables were analysed using descriptive analysis with mean and standard deviation, while frequencies and percentages were calculated for categorical variables. Chi-square test was used to investigate association between functional outcome and premorbid sociodemographic information, injury-specific data, as well as other relevant information post discharge. After the initial analysis, a multiple logistic regression analysis was used to determine the independent variables that most determined the outcome groups on the GOSE. Only variables significantly related to GOSE in the association study were included into the regression analysis. A p value of less than 0.05 was considered statistically significant.

The second phase was developing a prediction model. The prediction model for functional outcome was built by using multivariate logistic regression analysis to derive the best-fitting model. The model was then internally validated with split-sample cross-validation [28]. Performance of a statistical prediction model can be assessed in various ways; with performance quantified in terms of calibration using the Hosmer-Lemeshow “goodness-to-fit’ test, and discrimination with measures such as sensitivity, specificity and the area under the receiver operating characteristic curve [27]. In this study, calibration of the model was measured using the Hosmer-Lemeshow test. Discrimination of the derived models was measured using the C-statistics (equivalent to the area under the receiver operator characteristic curve) [27] which is the probability that a randomly selected patient who experienced the event of interest had a higher predicted probability than a randomly selected patient who did not experience the event. All statistical analysis was done using SPSS version 27 (IBM® SPSS® Statistics).

## Results

A total of 2699 medical reports of patients with mild to severe TBI secondary to RTA from 2009 to 2019 were analysed. From the total reports screened, 1939 patients fulfilled the inclusion criteria. Table 1 shows the characteristics of the 1939 patients who were included in the study.

**Table 1:** Socio-demographics and clinical background information of the participants and the association with functional outcome.

Variables	Frequency (n)	%	Good outcome n (%)	Poor outcome n (%)	p value
Age at accident (years)					
Less than 40	1395	71.9	482 (34.6%)	913 (65.4%)	<0.001
More or same as 40	544	28.1	105 (19.3%)	439 (80.7%)	
Gender					
Male	1608	82.9	470 (29.2%)	1138 (70.8%)	0.027
Female	331	17.1	117 (35.3%)	213 (64.7%)	
Ethnicity					
Malay	1308	67.5	449 (34.3%)	859 (65.7%)	
Chinese	208	10.7	43 (20.7%)	165 (79.3%)	<0.001
Indian	413	21.3	93 (22.6%)	319 (77.4%)	
Others	10	0.5	2 (20.0%)	8 (80.0%)	
Marital Status					
Single/ widowed	1164	60	373 (32.0%)	791 (68.0%)	0.037
Married	772	39.8	213 (27.6%)	559 (72.4%)	
Education level					
No education	143	7.3	24 (16.9%)	119 (83.1%)	
Primary	589	30.4	115 (19.5%)	474 (80.5%)	<0.001
Secondary	860	44.4	284 (33.0%)	576 (67.0%)	
Tertiary	347	17.9	164 (47.3%)	183 (52.7%)	
Main vehicle involved in RTA					
Car	243	12.5	85 (35.1%)	158 (64.9%)	
Motorcycle	1519	78.3	460 (30.3%)	1059 (69.7%)	0.083
Lorry	20	1.0	5 (25.0%)	15 (75.0%)	
Pedestrian	106	5.5	26 (24.5%)	80 (75.5%)	
Others	23	1.2	2 (8.7%)	21 (91.3%)	
Severity of TBI					
Mild	675	34.8	283 (41.9%)	392 (58.1%)	
Moderate	448	23.1	141 (31.5%)	307 (68.5%)	<0.001
Severe	659	34.0	107 (16.3%)	552 (83.7%)	
Neurosurgical Intervention					<0.001
No	1362	70.2	472 (34.7%)	890 (65.3%)	
Yes	576	29.7	115 (20.0%)	461 (80.0%)	
Presence of Concomitant injury					
No	268	13.8	77 (28.7%)	191 (71.3%)	0.55
Yes	1671	86.2	510 (30.5%)	1160 (69.5%)	
ICU admission					
No	1204	62.1	442 (36.7%)	762 (63.3%)	<0.001
Yes	735	37.9	145 (19.8%)	589 (80.2%)	
Headache					0.027
No	849	43.8	235 (27.7%)	614 (72.3%)	
Yes	1090	56.2	352 (32.3%)	738 (67.7%)	
Post trauma Seizure					<0.001
No	1739	89.7	571 (32.9%)	1168 (67.1%)	
Yes	200	10.3	16 (8.0%)	184 (92.0%)	
Behaviour Issue					<0.001
No	1140	58.8	467 (41.0%)	673 (59.0%)	
Yes	799	41.2	120 (15.0%)	679 (85.0%)	
Type of main behaviour issue					
No issue	1140	58.8	467 (41.0%)	673 (59.0%)	
Easily angry and agitated	335	17.3	59 (17.7%)	275 (82.3%)	<0.001
Withdrawn	119	6.1	22 (18.5%)	97 (81.5%)	
Child-like, or mixed	345	17.8	39 (11.3%)	306 (88.7%)	
Residence post injury					
Alone or with friends	127	6.5	79 (62.2%)	48 (37.8%)	
Immediate own family	847	43.7	239 (28.3%)	608 (71.7%)	<0.001
Parents	931	48	267 (28.7%)	664 (71.3%)	
Nursing home or residential care	33	1.7	2 (6.0%)	31 (94%)	
Acute significant medical inpatient complications					
No	1730	89.2	565 (32.7%)	1165 (67.3%)	<0.001
Yes	209	10.8	22 (10.5%)	187 (89.5%)	
Cognitive Impairments					
Severe	84	4.3	2 (2.4%)	82 (97.6%)	
Moderate	422	21.8	50 (11.9%)	372 (88.1%)	<0.001
Mild	479	24.7	150 (31.3%)	329 (68.7%)	
Normal	749	38.6	369 (49.3%)	380 (50.7%)	
Duration Post TBI					
Less than 2 years	1474	76.0	441 (29.9%)	1033 (70.1%)	
2 to 5 years	422	21.8	133 (31.5%)	289 (68.5%)	0.82
More than 5 years	43	2.2	13 (30.2%)	30 (69.8%)	
Household income pre-injury					
B40	1222	94.4	359 (29.4%)	863 (70.6%)	
M40	62	4.8	15 (24.2%)	47 (75.8%)	0.77
T20	10	0.8	3 (30.0%)	7 (70.0%)	

TBI, traumatic brain injury

RTA, road traffic accident

ICU, intensive care unit

AED, antiepileptic drug

B40, bottom 40%

M40, middle 40%; T20, top 20%

The mean age of the study participants was 32.4 ± 13.7 years. In terms of gender distribution, 83% of the patients were male, reflecting the similar TBI distribution worldwide. Malay ethnicity made up 67.5% of the study population, similar to Malaysian population. However, Chinese (11% in the study population versus 20.6% in Malaysia) and Indian (21% in the study population versus 6.2% in Malaysia) did not represent Malaysian population. Majority of the patients completed primary and secondary education and were employed during the injury. The overall mean duration post TBI was 19.4 ± 13.3 months. The mean duration of TBI among patients of less than 2 years injury was 13.6 ± 5.6 months.

Motorcyclist made up 78% of the TBI victims. Majority had extracranial concomitant injuries with facial injuries being the most common among all. Slightly more than half of the patients reported to have post-traumatic headache, while only about 10% of them have post traumatic seizures. Majority of the patients were from the poor socio-economic strata (94.4%) and falls into the B40 group of Malaysian household income group. Slightly more than half of the patients (57%) were able to return to driving, while 40% were able to return to work during the consultation.

Table 2 shows the distribution of patients in the GOSE categories in our study population. More than two thirds of the patients reported poor outcomes and 40% of them were in the severe disability categories. For patients who reported to have the good outcome, more than two-thirds (70%) of them were in the lower good recovery category. This category represents a group that can return to work but may have some difficulties to resume regular social and leisure activities outside home and suffers occasional strain in family and friendship. Sub-group analysis of patients who suffered the TBI of more than 5 years showed similar GOSE breakdown, with 30.2% of them reported good outcome.

**Table 2:** Functional outcome at based on the Glasgow Outcome Scale-Extended (GOSE).

Outcome Variable (GOSE)	n	Percentage (%)
Good Outcome (category 7–8)	587	30.3
7 –Lower Good Recovery	406	21.0
8 –Upper Good Recovery	181	9.3
Poor Outcome (category 1–6)	1352	69.7
1 –Dead	0	0
2 –Vegetative state	28	1.4
3 –Lower Severe Disability	265	13.7
4 –Upper Severe Disability	307	15.8
5 –Lower Moderate Disability	470	24.2
6 –Upper Moderate Disability	282	14.6

Univariate analysis of the data identified fourteen factors that were significantly affecting the functional outcome of patients post TBI. Factors significantly associated with functional outcome include age, gender, ethnicity, marital status, education level, severity of brain injury, neurosurgical intervention, ICU admission, presence of inpatient complications, cognitive impairment, post-traumatic headache, post traumatic seizures, significant behavioural issue; and residence post discharge (p<0.05). Duration post TBI during the review, presence of concomitant injuries, main vehicle involved in RTA and household income pre-injury were not found to be significantly associated with functional outcome. Multivariate binomial logistic regression was performed to ascertain the association between the fourteen factors with the likelihood of good or poor functional outcome after TBI. The logistic regression model was statistically significant with p<0.05 with a nonsignificant Hosmer and Lemeshow test. The model explained 32% (Nagelkerke R2) of the variance in functional outcome. After adjusting for the confounding factors, seven of the factors were found to significantly predict outcome post TBI, including age at accident, severity of TBI, neurosurgical intervention, cognition impairment measured by MMSE, post traumatic seizure, behavioural issue and residence post trauma (Table 3).

**Table 3:** The multivariate logistic regression analysis between premorbid socio-demographic data, injury details and post injury complications with good functional outcome based on Glasgow Outcome Scale-Extended (GOS-E).

Independent variables	Odds ratio	95% Confidence Interval	p value
Age group			
≥40 years old	1		
<40 years old	2.25	1.59–3.16	**<0.001**
Severity of traumatic brain injury (TBI)			
Severe TBI	1		
Mild TBI	2.14	1.57–2.92	**<0.001**
Moderate TBI	1.73	1.24–2.41	**0.001**
Neurosurgical Intervention			
Yes	1		
No	1.35	1.01–1.81	0.04
Post Traumatic Seizure			
Yes	1		
No	2.67	1.46–4.90	**0.001**
Behavioural issue			
Yes	1		
No	3.13	2.40–4.10	**<0.001**
Cognitive Impairment			
Moderate & Severe	1		
Mild	1.65	1.24–2.20	**<0.001**
Normal	4.81	3.27–7.00	**0.001**
Residence post injury			
Nursing home	1		
Staying alone or with friends	7.06	1.33–37.48	**0.02**
Staying with immediate family (spouse and/or children)	3.97	0.778–20.256	0.097
Staying with parents	2.69	0.53–13.622	0.23

A prediction model for functional outcome was built using the results from the multivariate logistic regression analysis. Based on the predictive model, younger age less than 40, mild brain injury, absence of cognitive impairment, absence of post traumatic seizure, absence of post traumatic behavioural issue and staying alone or with friends post injury are significant predictors of good functional outcome post TBI.

**Fig 1:**
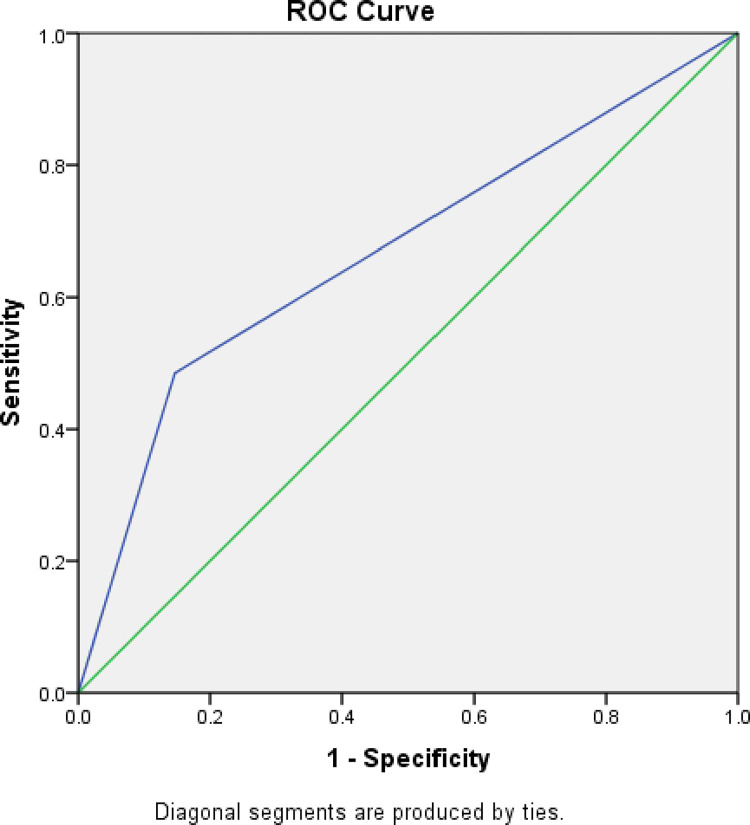
Receiver operating characteristic curves in the derivation data set. AUC indicates the area under the curve, and CIs, confidence intervals.

**Fig 2:**
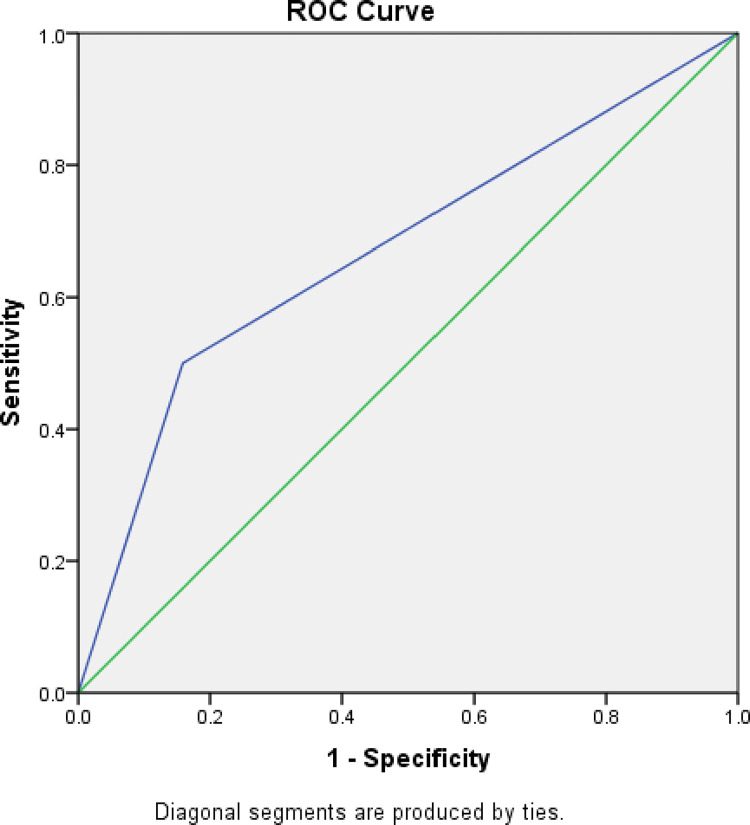
Receiver operating characteristic curves in the validation data set. AUC indicates the area under the curve, and CIs, confidence intervals.

The obtained model was then validated internally using 70:30% split-sampling validation method. The AUC of ROC, also called the C-statistic was calculated. When testing the performance of the predictive model, the median ROC value was 0.67 (range 0.63–0.71) with p value of less than 0.001. (Figs 1 and 2). This implied that the model generated has acceptable discrimination, moderately accurate and better than chance alone in predicting functional outcome post TBI. The median p value for the Hosmer-Lemeshow goodness-of-fit test was 0.46.

## Discussion

This study examines the long-term outcome of patients with TBI from RTA ranging from an average of 19 months to almost 8 years post injury. We found that good outcomes were only reported in about 30% of our study population. The rate was similar regardless of the duration after TBI, whether less or more than 5 years post injury. Since we only examined reports from patients who survived the RTA, the rate would probably be lower if we included ‘death’ as one of the poor outcomes. One of the criteria for good outcome was the ability to return to work, which is known to be heavily influenced by pending insurance claims. There was no local data available on this relationship in Malaysian population. Given that Malaysia is an LMIC, and most of our patients were from the lower income group involved in physical work, minor physical and cognitive disabilities themselves may greatly affect the patients’ ability to return to work. In general, the good outcomes were lower than most studies in the developed countries which reported favourable outcomes at a rate of 40–50% [6–8, 29].

One of the possible reasons for a higher proportion of favourable outcome in developed and HIC is the better medical services after acute TBI, and a more coordinated and long-term rehabilitation program. We were unable to examine the role of rehabilitation care in our study population because of the limited information in the reports. Additional data which may have significant effects on the functional outcome such as the time to first intensive rehabilitation care [30], frequency of therapy and duration of rehabilitation, were not captured in most of the reports. Rehabilitation services after TBI in Malaysia only started to expand steadily over the last 8 years, with at least one hospital in each state provides an inpatient multidisciplinary rehabilitation program. However, the number of trained staff and beds available for patients undergoing rehabilitation are limited in most of the states. For example, Kelantan, one of the states in east coast Malaysia has less than 20 inpatient rehabilitation beds for the whole state of two million people. Most patients may not receive the optimal acute inpatient rehabilitation care and have shorter duration of follow-up after discharged. We can assume that majority of the study population received a similar rehabilitation care post TBI, and our result reflects the average care in Malaysia.

Neurosurgery and neurotrauma services in Malaysia on the other hand, has been rapidly developing due to the exponentially increasing demand for the care of trauma patients [31]. Currently there are neurosurgical services in almost every state in Malaysia to cater for the increasing number of patients. The services are found in the public hospitals, university hospitals and some private hospitals [31]. Despite the expansion of neurosurgery services here, there is still relatively low neurosurgeon to population ratio with less optimum access to neurosurgical services in certain rural regions [32]. Patients presented to hospital in rural areas will need to be referred to hospital with neurosurgery facilities, hence there may be delay in time to treatment.

Another possible explanation for the lower rate in our study is the differences in methods of data collection that influence the differences in outcome reporting. We use a stricter criterion of categorization for good functional outcome. Only Level 7 and 8 of the GOSE scale (the ‘good recovery’ categories) were considered as having good outcome, while some studies have included level 5 and 6 (‘moderate disability’ categories) as ‘favourable’ outcomes [3, 33, 34]. We chose to define the good outcome this way to ensure that all patients categorized in the group have a significant degree of re-establishment of meaningful abilities and reengagement in life activities without assistance. It is also easier for us to compare with the outcomes previously studied in Malaysian population using similar criteria [21, 30, 35]. On the other hand, most studies also included death as part of poor outcomes (Level 1 of the GOSE scale).

A few other studies conducted in LMIC have reported a similar lower proportion of good outcome. For example, only 20% of TBI patients in Sri Lanka attained an overall good recovery (GOSE 7 or 8) at 6 months follow up [13]. In another study conducted on severe TBI patients in Thailand, the authors reported that only one-third of the patients who still came for hospital follow-up at one year, attained good recovery [12]. Another centre in India reported a slightly higher rate of good outcome with 45%, but the authors included paediatric population in the study and reported good outcome from both good recovery and moderate disability groups [36]. The authors, however, did not mention the time of outcome assessment. A retrospective study performed in Lombok, Indonesia reported a different finding with a high proportion (63%) of patients showing good outcome [37]. In this study, the authors too, did not state the time when the functional assessment was made or the detailed care that the patients received. In most of these studies conducted in LMIC, limited resources for rehabilitation care and the high number of defaulted follow-ups were highlighted. Similarly, motorcyclists were also the most commonly involved in RTA leading to TBI in all these countries.

Since Malaysia is a multi-ethnic society in Southeast Asia, we were also interested to examine the role of ethnicity on the outcome of these patients. Majority of the patients were of Malay ethnicity; and this is representative of the population distribution in Malaysia whereby Bumiputera (Malays and indigenous peoples) made up about 63% of the Malaysia population [38]. However, the proportion of Chinese and Indian patients in this study did not reflect the Malaysian distribution. There was a significant difference in the functional outcome reported among the different ethnicities, with Malays showing a statistically higher rate of good recovery. However, ethnicity was not found to be a significant risk factor for outcome after removing the confounders. This outcome contrasts with previous studies performed in the United States, where ethnicity remains a strong independent risk factor for trauma mortality and poor outcome [39, 40].

In previous studies, a decline in functional status was reported with longer duration post TBI [8, 9]. There were more patients who reported a drop in the GOSE category between 5 to 10 years follow up, with one third of them reported a decline after 10 years [8]. We did not observe the different proportion of lower functional status in our patients that have TBI for longer than 5 years. Duration of TBI was also not a significant factor predicting outcome in this study, with similar rates of good and poor outcomes despite the duration post injury. However, since this is not a cohort study and the information was obtained at a single assessment, we could not ascertain whether there was a change in the GOSE categories with time.

Residence after TBI can have broad implications. The ability of independent living was shown to be associated with psychosocial and emotional sequelae of individuals with TBI [41]. These could affect the functional outcome and social integration of the patients. In most developed countries, a high number of those living alone prior to injury returned to premorbid state of living by one year and remained so up to five years post injury [42, 43]. We did not have the Malaysian data on the change of residential status post injury but in general, most TBI patients moved back to their parents or siblings’ homes. This was more common in unmarried patients with moderate to severe injuries. Parents or immediate family members tend to shoulder the responsibility as primary caregivers for a long period of time [44]. Our study found that independent living either alone or with friends is an independent predictor of good functional outcome. This finding further emphasizes the importance of independent living care rehabilitation programs among suitable patients post TBI in this country.

We have also developed and internally validated a prediction model for good functional outcome. This prediction model identified six factors, namely less than 40 years old, mild TBI, absence of cognitive impairment, significant behavioural issue, or post traumatic seizure, as well as independent living to predict good outcome post TBI. The model demonstrated acceptable discrimination in both the derivation and validation cohort (AUCs = 0.67). Our results are particularly relevant for TBI patients in this region after surviving the initial trauma and can serve as a guide on management decisions, information on outcome expectation, selection of patients for rehabilitation, as well as improvement on effectiveness of resource utilization [45].

The limitations of working with a retrospective data, especially with a single time point assessment, include the inability to determine the progress or decline of functional outcome over time. Moreover, this study population is biased towards a group of patients who were involved in insurance claimant and litigation purposes for either loss of dependency or injury claims or both, after RTA. We also did not analyse the proportion of motorcyclists wearing helmets and its relationship with TBI outcome. It is mandatory to wear helmets in Malaysia and the number of patients not wearing helmet would have been negligible.

Nevertheless, the strengths of this study include the consistency of the reports because they were written by one senior clinician who acted as an independent assessor of both complainant and defence. Bias in reporting the deficits was low because the assessor was not the only clinician who assessed the patients and there was a high possibility for the reports to be presented in court. This study also has a large sample size of patients from all over Malaysia. The development of a prediction model and internal validation of the model is hoped to add benefit to clinicians in their clinical decisions in developing the optimal rehabilitation program to better maximize functional outcome among TBI survivors. For future research, the predictive model should undergo an external validation in other populations and clinical settings.

### Conclusion

In conclusion, patients with TBI from RTA have a slightly low rate of good functional outcome. Due to the selected TBI population analysed, caution must be made when interpreting the finding to general population. The factors identified for a good functional outcome in the prediction model are comparable to other studies worldwide. These findings highlight the need to address the care after TBI, especially the optimal management and rehabilitation of the modifiable factors to achieve better functional outcome.

## Supporting information

S1 File (DOCX)

S1 Raw data (XLSX)
