# Supplementary material for: Outcome of 1939 traumatic brain injury patients from road traffic accidents: Findings from specialist medical reports in a low to middle income country (LMIC)
Source: PLoS One. 2023 Sep 13;18(9):e0284484. doi: 10.1371/journal.pone.0284484 (PMC10499241; doi:10.1371/journal.pone.0284484)
Supplement: S1 File — (DOCX) [file pone.0284484.s001.docx]

| **Statistic** | **Value** | **95% CI** |
| --- | --- | --- |
| Sensitivity | 55.65% | 46.09% to 64.91% |
| Specificity | 75.22% | 70.33% to 79.67% |
| Positive Likelihood Ratio | 2.25 | 1.76 to 2.87 |
| Negative Likelihood Ratio | 0.59 | 0.48 to 0.73 |
| Positive Predictive Value | 42.67% | 36.80% to 48.75% |
| Negative Predictive Value | 83.65% | 80.52% to 86.37% |
| Accuracy | 70.35% | 65.95% to 74.48% |

ROC for modelling data


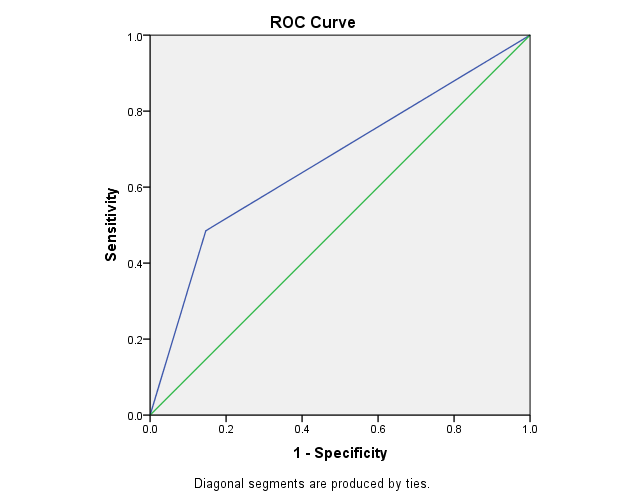


| **Area Under the Curve** | | | | |
| --- | --- | --- | --- | --- |
| Test Result Variable(s): Predicted group | | | | |
| Area | Std. Error^a^ | Asymptotic Sig.^b^ | Asymptotic 95% Confidence Interval | |
|  |  |  | Lower Bound | Upper Bound |
| .669 | .018 | .000 | .634 | .705 |
| The test result variable(s): Predicted group has at least one tie between the positive actual state group and the negative actual state group. Statistics may be biased. | | | | |
| a. Under the nonparametric assumption | | | | |
| b. Null hypothesis: true area = 0.5 | | | | |

| Area | Std Error | Asymptotic Sig | Asymptotic 95% Confidence |  |
| --- | --- | --- | --- | --- |
|  |  |  | Lower bound | Upper bound |
| 0.67 | 0.018 | 0.000 | 0.63 | 0.71 |

ROC for cross validation sample


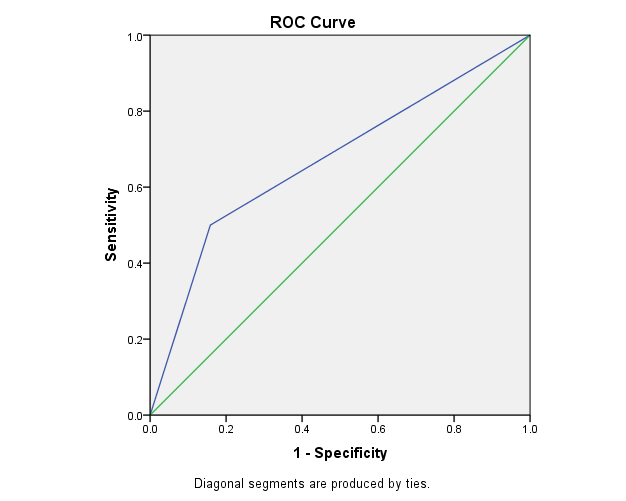


| **Area Under the Curve** | | | | |
| --- | --- | --- | --- | --- |
| Test Result Variable(s): Predicted group | | | | |
| Area | Std. Error^a^ | Asymptotic Sig.^b^ | Asymptotic 95% Confidence Interval | |
|  |  |  | Lower Bound | Upper Bound |
| .671 | .028 | .000 | .615 | .726 |
| The test result variable(s): Predicted group has at least one tie between the positive actual state group and the negative actual state group. Statistics may be biased. | | | | |
| a. Under the nonparametric assumption | | | | |
| b. Null hypothesis: true area = 0.5 | | | | |

| Area | Std Error | Asymptotic Sig | Asymptotic 95% Confidence |  |
| --- | --- | --- | --- | --- |
|  |  |  | Lower bound | Upper bound |
| 0.67 | 0.028 | 0.000 | 0.62 | 0.73 |
